# Supplementary material for: Spatiotemporal Variation in Ground Level Ozone and Its Driving Factors: A Comparative Study of Coastal and Inland Cities in Eastern China
Source: Int J Environ Res Public Health. 2022 Aug 5;19(15):9687. doi: 10.3390/ijerph19159687 (PMC9367812; doi:10.3390/ijerph19159687)
Supplement: Supplementary file 1 [file ijerph-19-09687-s001.zip › ijerph-1827867-supplementary.pdf]

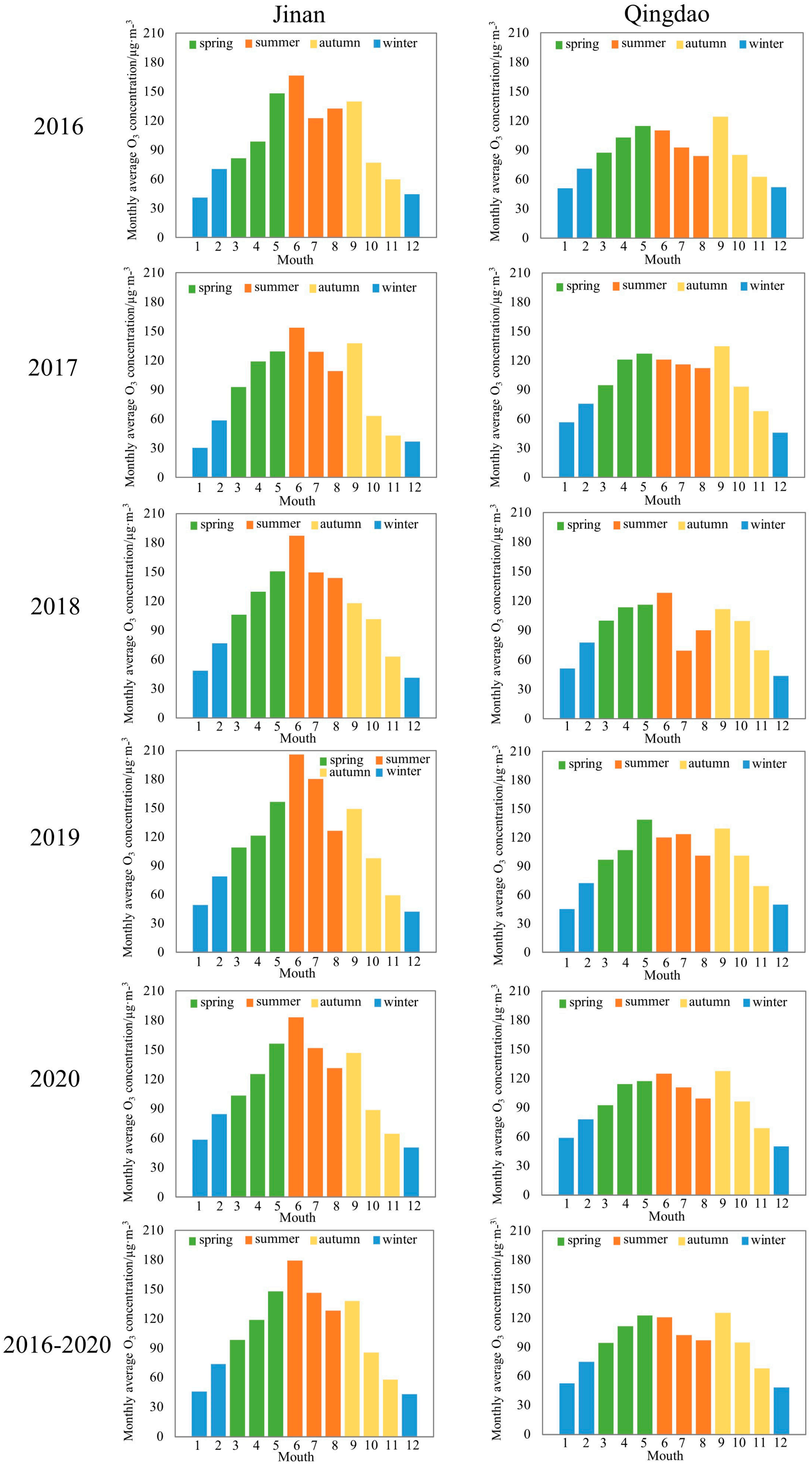

Supplementary Figure S1 Histogram of monthly average O_3 concentration changes from 2016 to 2020.

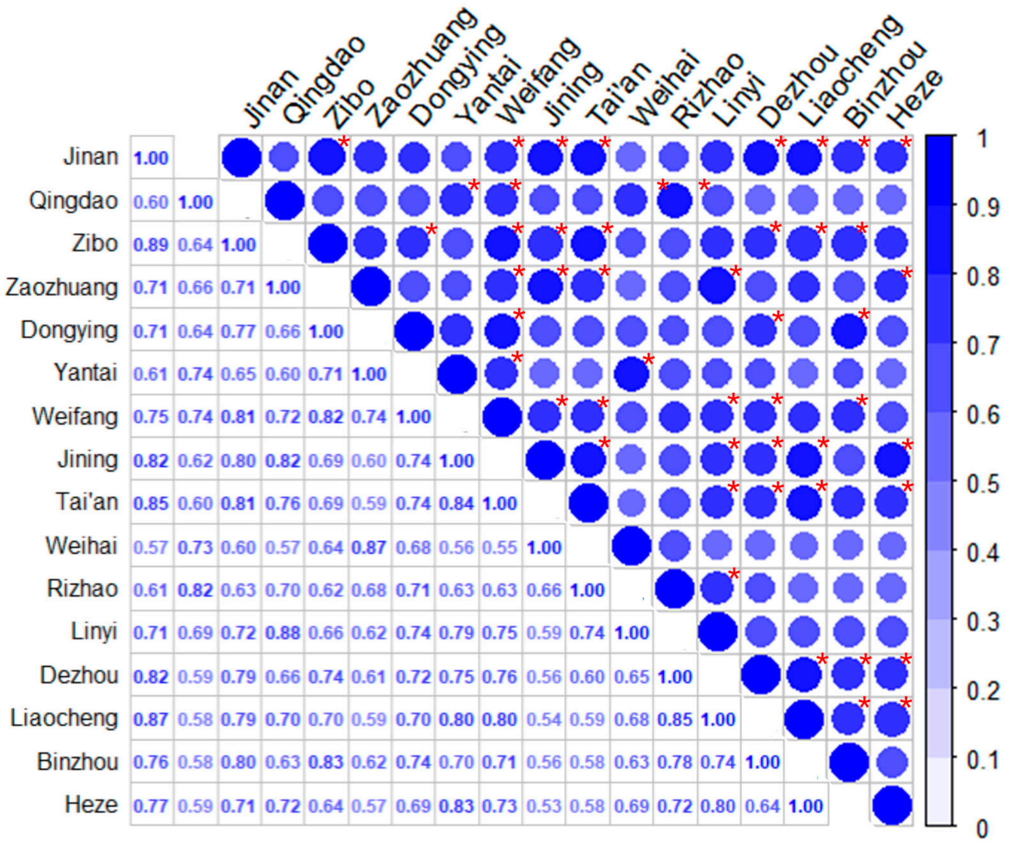

Supplementary Figure S2 ARsq among cities in Shandong province from 2016 to 2020. * is indicates passing the 95% significance level test.

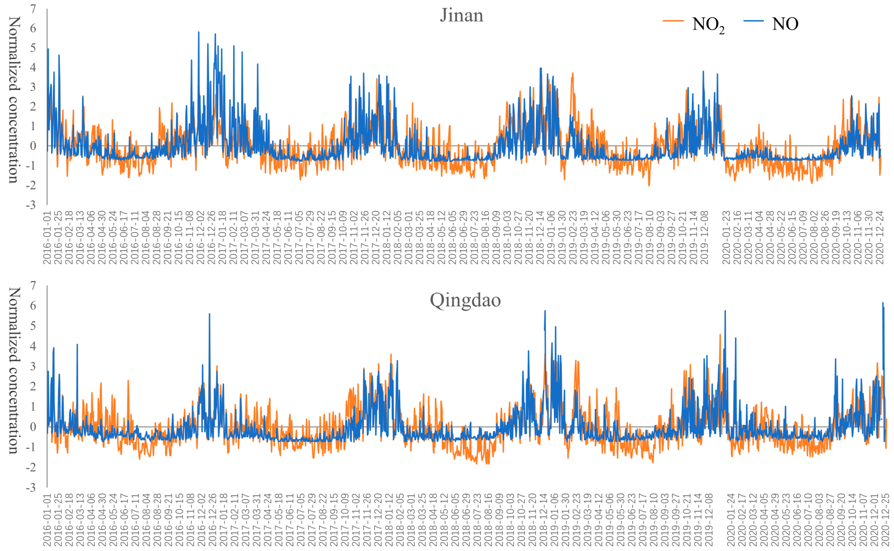

Supplementary Figure S3 Line chart of the daily change of the daily maximum 8-hour average (DMA8) O3 concentration in Jinan and Qingdao.
